# 507. Systematic Review of Erosive Pustular Dermatosis in Burns: An Underrecognized Cause of Delayed Wound Healing

**DOI:** 10.1093/jbcr/irag033.166

**Published:** 2026-04-06

**Authors:** Smiti Gandhi, Marie Vu, Ria Kanotra, Michael Wilkerson

**Affiliations:** University of Texas Medical Branch, John Sealy School of Medicine, Houston, TX; University of Texas Medical Branch, Department of Dermatology, Galveston, TX; University of Texas Medical Branch, John Sealy School of Medicine, Houston, TX; University of Texas Medical Branch, Department of Dermatology, Galveston, TX

## Abstract

**Introduction:**

Erosive pustular dermatosis (EPD) is a rare inflammatory disorder characterized by crusts, erosions, and pustules on atrophic skin. It typically arises in adults aged 60-70 and often involves the scalp. Lesions may be disfiguring and persist or relapse despite therapy. Long-term complications include alopecia, atrophy, and telangiectasia, particularly when diagnosis and treatment are delayed. Common risk factors include trauma or surgical procedures such as Mohs micrographic surgery.

**Methods:**

This review focuses on EPD in burn patients and how its presentation compares to classical scalp-limited disease. Advanced search queries of “burn(s)” OR “burn patients” AND “EPD” were performed on Google Scholar, PubMed, Scopus, and JAAD.

**Results:**

11 studies comprising 20 patients (aged 2-66 years; mean: 40 years; 55% male) were included. Burn etiologies included flame, gas, scald, boiling water, chemical, sun, and self-inflicted injury. Among 10 cases with available data, mean total body surface area (TBSA) was 23% (range 2-60%). While the scalp is most involved in classical EPD, burn-associated cases can also present at other sites. Lesions were often initially characterized as “non-healing” burns and were sometimes complicated by *Staphylococcus aureus* superinfection. Clinically, patients developed erythematous plaques with hyperkeratotic crusts, erosions, and pustules, while biopsies demonstrated neutrophilic infiltration. Treatment generally included various combinations of high-potency topical corticosteroids (most often clobetasol), oral steroids, and dressing changes, which often led to stabilization, improvement, or resolution.

**Conclusions:**

Burn-associated EPD differs from classical disease by affecting younger patients. It arises from diverse etiologies.

**Applicability of Research to Practice:**

Early recognition of EPD in burn patients is essential to prevent misdiagnosis, avoid unnecessary antimicrobials, and improve outcomes.

**Funding for the study:**

N/A.

